# Supplementary material for: Are carbon emissions trading and green financial instruments synergistic? -Comprehensive quantitative research based on content analysis
Source: PLoS One. 2024 Mar 7;19(3):e0298601. doi: 10.1371/journal.pone.0298601 (PMC10919601; doi:10.1371/journal.pone.0298601)
Supplement: S1 Data — (ZIP) [file pone.0298601.s001.zip › Data/地级市Z_F工作报告.zip!/░▓╗╒/╗┤▒▒╩╨/└┤╫╘╣1⁄2╓┌║┼í╛┬φ┐╦╩2╛▌═°í┐_2012.docx]

各位代表：

    现在，我代表市人民政府，向大会报告政府工作，请予审议，并请市政协委员和其他列席人员提出意见。

****一、开局之年：转型发展迈出坚实步伐****

    2011年是“十二五”开局之年，也是经济形势复杂多变、各种矛盾集中凸显的一年。面对宏观政策趋紧和城市转型双重考验，在省委、省政府和市委的坚强领导下，在市人大、市政协的监督支持下，市政府团结带领全市人民，深入贯彻落实科学发展观，以转型发展为主线，以改革创新为主动力，以“大投入、大招商、大建设、大发展”为主抓手，着力保增长调结构，打基础增后劲，惠民生促和谐，胜利完成市十四届人大四次会议确定的年度目标任务。

   初步核算，2011年全市实现地区生产总值560亿元，比上年增长12.5%；财政收入90亿元，增长28.6%；固定资产投资445亿元，增长30%以上；规模以上工业增加值420亿元，增长15.5%；社会消费品零售总额148亿元，增长18%；进出口总额2.4亿美元，增长21%；城镇居民人均可支配收入17600元，增长16%；农民人均纯收入6380元，增长19.5%。全市经济、社会、文化、生态文明建设取得新进展、出现新变化、呈现新亮点。

    一是质量效益大幅提高。工业经济提质增效，全年新增规模以上工业企业125家，总数达到524家；新增销售收入亿元以上企业123家，增长1.5倍。规模以上工业总产值、主营业务收入双双首次突破千亿元大关，利税再次突破百亿元大关，分别达到1147亿元、1430亿元和138亿元，总量、增幅均排名全省前六位；工业产品销售率位居全省第一，企业经济效益综合指数创历史新高。产业结构逐步优化，煤电和非煤电产业增加值分别达到250亿元、170亿元，增长5%和39%，非煤电产业增加值所占比重比上年提高8个百分点。县区经济快速崛起，县区和市开发区实现生产总值341亿元，均增长18%以上，占全市总量的60.9%。新兴产业发展迅猛，工业总产值增速居全省第2位。

    二是动力活力明显增强。科技创新能力进一步提高，新增国家级高新技术企业5家、省级企业技术中心4家、省级新产品20个，高新技术产业总产值突破100亿元，增加值增速居全省第3位；龙波电气、天地人荣获“中国驰名商标”称号；我市跻身“全国科技进步先进市”行列。扎实开展“招商引资推进年”活动，加强经济合作与交流，与巴西克里西乌马市达成友好城市意向，与美国阿罕布拉市签署了友好交流城市协议，成功举办第二届淮海经济区核心区市长会议。第六届中国（淮北）煤矿机械博览会成果丰硕。全年累计引进内资375亿元，增长33.5%；实际利用外资2.8亿美元，增长46%。引进国内500强企业2家，引进亿元以上企业30家。着力推动全民创业，加快发展非公经济，全市新增私营企业1299户、个体工商户5228户，非公经济所占比重比上年提高1个百分点。扩权强镇试点改革全面推进，事业单位机构编制清理规范、人事制度改革取得积极进展。

    三是后劲潜力正在积蓄。着力扩大有效投入，全市新建、续建重大项目225个，其中亿元以上工业项目133个，完成工业投资265亿元，占全市固定资产投资的60%。思朗食品、中臣型钢等一批项目竣工投产，袁店一井、袁店二井、杨柳、青东4对矿井建成投产，全省首座110千伏智能变电站建成，大唐虎山电厂、中利电厂二期、国购城市综合体等130个项目开工建设，翔宇物流、淮北汽车城二期等商贸物流项目建成运营。平山电厂项目历经8年终获国家发改委核准建设，淮水北调工程通过水利部专家评审。

    四是载体平台逐步夯实。抢抓机遇，适时启动市开发区新区建设。规划面积30平方公里的市开发区新区获得省政府批准，累计完成投入11.3亿元，其中基础设施投入2亿多元。4个月时间全面完成1674户征收工作，89栋安置房主体工程施工过半。一期5.26平方公里“九通一平”加速推进，梧桐大桥、吉山路大桥开工建设，二期14.74平方公里建设已经启动。市开发区龙湖项目区投入2亿元加强基础设施建设，引进项目36个。临涣工业园高起点编制了园区空间发展规划和产业规划，构建了园区管理体制和运行机制，考伯斯等下游产业项目正在积极洽谈推进。县、区开发区完成基础设施投资6.5亿元，承载能力进一步增强；理士工业园、徽钢铜业、宝博合成革等一批项目落户，凤凰山工业园获“全国农产品加工示范基地”称号。

    五是创城水平显著提升。去年是创城工作重视程度最高、资金投入最大、群众受益最多、社会参与最广的一年，全年累计完成城市基础设施投资18亿元，我市荣获“全国文明城市提名奖”和“第二届全省文明城市”。投入3亿多元，完成75个老旧住宅小区综合整治；投资4170万元，建成桓谭公园、时代公园“清风苑”；投资1030万元，实施道路、公园、广场绿化，人居环境进一步优化。投资6亿多元，淮海东路、沱河路等15条骨干路网开工建设，方安路、古城东路、惠苑东路、南湖路北段建成通车，梧桐北路、宿丁路全面改造完成；投资4369万元，铺设人行道15.2万平方米，人民路、相山路等10余条主干道全部改造为沥青路面；投资1796万元，铺设供水、供气管网41.8公里，城市承载能力进一步提高。积极推行社区“网格化”管理，加强市容环境综合整治，实施城市亮化工程，开展文明交通行动，城市形象进一步提升。大力弘扬“燃烧自己、温暖他人”的煤城精神，姚勇等72名同志入选“中国好人榜”，市民素质进一步提高。成功举办“激情广场·爱国歌曲大家唱”大型歌咏活动、“全国道德模范与身边好人现场交流活动”，《人民日报》等11家主流媒体对我市进行集中宣传，淮北的知名度、影响力大大提升。

    六是城乡统筹步伐加快。坚持以工促农、以城带乡，不断加大强农惠农富农力度，市财政“三农”支出8亿元，增长10%。扎实开展“小麦千斤市”创建活动，实施玉米振兴计划，累计投入抗旱资金2亿元，大旱之年粮食生产实现“八连丰”，总产达126万吨。不断加大结构调整力度，全年新增规模以上土地流转面积3.4万亩，新增设施蔬菜面积1.7万亩，扩建标准化养殖小区（场）35家，新增规模以上农业产业化龙头企业5个，农村专业合作经济组织发展到662家。深入推进城乡一体化，试点范围扩大到“一区两办十镇”，建成、在建集中居住区52个。重点支持城镇和集中居住区基础设施建设项目32个。完成农田水利基本建设投资9813.5万元，王引河下段、懈河洼地治理、华家湖水库防渗整治工程全面完工。改造县乡村道路116公里。农村清洁工程连续两年全省领先。

    七是民生工作成效显著。民生工程全部完成年度任务，累计投入18.4亿元。生活保障类项目扩面提标。率先在全省提高城乡低保标准和五保供养标准，城市低保标准由每人每月246元提高到290元，农村低保标准由每人每年1000元提高到1260元，五保集中供养标准从每人每年1900元提高到3500元，5.3万名农村低保户和五保户基本生活得到保障。城镇退役士兵自谋职业补助标准由每人2.3万元增加到4.5万元。3400套廉租住房和7000套公共租赁住房开工建设。教育培训类项目有序开展。落实义务教育保障经费1.6亿元，惠及25.9万名学生。新增职业技能教育资助项目，由市财政投入1200多万元，对我市职业学校淮北籍学生每人每年资助1000元。资助高校、中职和普通高中家庭经济困难学生2.4万名，加固重建校舍29.3万平方米。医疗卫生工程扎实推进。城乡居民医疗保障覆盖率超过95%，122万人参加新农合、52万人参加城镇居民基本医疗保险。完成免费婚检3.5万人，补助农村孕产妇住院分娩1.6万人，免疫规划接种率达到90%以上。农业和农村基础设施类工程全面完成。实施“一事一议”财政奖补试点项目270个，解决了7.4万农村人口饮水安全问题，15座农村公路危桥改造工程全部竣工。农村文化建设类项目力度加大。150个农家书屋、4所乡镇综合文化站建成投入使用。家电下乡和家电以旧换新政策全面实施。在全国城市分类优势排行榜中，我市居民幸福感指数位居全省之首。

    八是社会事业长足进步。全年财政投入社会事业64.8亿元，占全市财政支出的76%。市一中第三次荣登“中国百强名校榜”，投资2.7亿元的市实验高中新校区投入使用。出台《淮北市学前教育三年行动计划》，新建、改建幼儿园23所。成立市职业教育工作委员会和职业教育局，职业教育园区即将开工。新体育场、市矿山博物馆建成使用，市图书馆、文化馆、方志馆免费向市民开放。柳孜运河码头遗址和汴河遗址百善老街及柳孜码头段进入申报世界文化遗产预备名单。扎实推进基层医疗卫生体制改革，39家基层医疗机构实施基本药物制度和药品零差率，307所村卫生室实行一体化管理。市人民医院被安徽省确定为区域性医疗中心。群众性体育活动蓬勃开展，竞技体育取得新成绩。人口和计生工作完成省下达指标。进一步加强环境保护工作，众城水泥纯低温余热发电等5个国家节能奖励项目、濉溪县污水处理厂等4个水污染减排项目投入运营，节能减排完成省下达控制指标。

    九是和谐局面更加巩固。社会治安综合治理进一步加强，群众安全感指数连续5年皖北第一，信访维稳工作全省领先。司法所和社区矫正基地建设基本完成。“六五”普法全面启动。高度重视食品药品安全监管，扎实开展地沟油和食品非法添加剂专项行动。深入实施机动车辆超限超载专项治理，在全省率先实现全面治超，从根本上遏制了公路超限超载运输现象。积极做好护校护园安保工作。加强安全生产监管，安全生产形势持续稳定好转。民族团结、宗教和睦局面得到巩固。创建全国无障碍建设城市通过国家验收。国防动员、人防和双拥优抚工作深入开展。机关事务管理、档案、外事、对台、地方志、保密工作不断加强，地震、气象、防灾减灾、应急管理工作扎实推进，妇女、儿童、老龄、残疾人事业实现新发展。

    十是政府建设得到加强。坚持科学理政、依法行政，加快推进政府职能转变和管理创新，加强效能建设和目标管理，自觉接受人大法律监督、工作监督和政协民主监督，加强与各民主党派、工商联、无党派人士协商，进一步密切与工会、共青团、妇联等人民团体的联系，认真听取各方面意见和建议，办理省、市人大代表议案、建议94件、政协委员提案171件，满意度98%以上。深入推进政务公开，支持网络问政，办理市长信箱、网民留言等3305件，办结率97%以上，人民网网民留言办理工作获全国先进。扎实做好政风行风热线工作，开设《说办就办》栏目，解决群众反映问题2693个。加强领导干部经济责任审计和专项审计，坚持不懈开展反腐倡廉工作，严肃查处违纪违法案件和失职渎职行为，维护了风清气正的政治生态环境。

    回顾过去一年的工作，我们不断加大改革创新力度，思路进一步拓宽，体制机制进一步完善，有效解决了前进中的困难和问题。

    一是完善机制真招商。出台《关于切实做好2011年招商引资工作的意见》，设立五个区域招商分局，组建五大产业招商中心，强化驻点招商、产业招商、以商招商。完善招商引资认定、考核、调度、奖惩兑现等工作机制，推行招商引资项目一会准入、一次性告知、一站式审批、一个窗口收费、一次性验收、一次性兑现“六个一”工作机制，对重大招商项目实行“一事一议、一企一策、特事特办”，全市上下真招商、招大商的热情高涨，一大批项目陆续签约落地。

    二是金融创新见成效。科学预判、远谋近施，独立设置了市金融办，出台了一系列鼓励金融机构支持地方发展的政策措施，淮北农村商业银行、马鞍山农商行濉溪支行正式开业，濉溪县信用联社改制正在加紧推进，新增小额贷款公司9家、担保公司6家。全年新增贷款105亿元，增幅居全省第2位，存贷比达到56.4%，比上年提高2.8个百分点。成立市上市办，支持企业上市融资，淮矿股份、口子酒业等10家企业进入上市辅导和培育期。成立市政府投融资管理委员会和管理中心，重组市建投集团，全年融资31.3亿元。国家农发行23亿元农村城镇化贷款项目获批，国开行20亿元保障性住房建设资金开贷，成功发行12亿元企业债券，设立了注册资本3.1亿元的城市转型风险投资基金，融资工作走在全省前列。

    三是城市建设有新招。实行市、县区一级规划、分级建设，出台《淮北市城市控制性详细规划技术通则》，切实维护规划的统一性、严肃性。坚持依法依规节约集约用地，成立市土地管理委员会，强化用地规划和计划管理，推行项目用地预审会审制度和履行保证金制度，依法分类处置闲置土地，多渠道解决用地难题，全年获批建设用地1.9万亩，收储土地1.1万亩。严格实行国有土地公开招拍挂制度，全年实现土地出让价款54.5亿元，增长59.3%。成立市重点建设局，全面实施规划、房屋征收、招投标、建设、审计等“八分开”，出台《淮北市建设工程施工招投标有效最低价评审暂行办法》，加强政府性投资项目审计，进一步完善了城市建设管理体制，提高了资金使用效率。

    四是帮扶企业出实策。设立“经济110”，组织开展“百名干部驻点帮扶百家企业”活动，积极帮助企业解决生产经营难题。成立企业办证巡回服务团，为62家企业一次性办理了土地证、房产证。成立中小企业融资服务中心，出台中小企业助保金贷款管理办法，推出企业动产抵押，股权、商标权质押贷款等创新性融资方式。全年中小企业贷款达133.2亿元，比年初增加30.7亿元。中小企业产值、税收分别增长26.5%、24%。出台了《市委、市政府关于推进口子酒业加快发展的若干意见》，支持口子酒业建设“一企三园”，打造百亿元企业。成功举办安徽酒类产品推介会，推出三十年型口子窖酒。去年口子酒业上交税收7.5亿元，在全省民营企业中名列第一。

   各位代表！回顾过去的一年，我们取得的成绩令人振奋，积累的经验弥足珍贵，创新的实践影响深远。在此，我代表市人民政府，向全市广大工人、农民、知识分子、干部和离退休老同志，向各民主党派、工商联、各人民团体和社会各界人士，向驻淮解放军指战员、武警官兵和政法干警，向中央和省驻淮单位，向关心和支持淮北发展的朋友们，向在我市创业的投资者、建设者表示衷心的感谢！

    在看到成绩的同时，我们也清醒地看到，全市经济社会发展还存在一些不容忽视的问题。总量不大、增速不快、结构不优仍是我们面临的最大问题；有效投资不足、城市严重缺水、发展空间受限既是现实问题又是长远问题；企业数量少、规模小、实力弱，城乡居民收入增长不快是长期困扰我市加快发展的关键问题；少数部门服务意识不强，作风漂浮、扯皮推诿，官僚主义和消极腐败现象依然存在。以上这些都是我们必须正视、需要努力克服的困难和问题。

****二、新的一年：转型发展步入攻坚阶段****

    2012年，是全面贯彻省第九次党代会和市第七次党代会精神的第一年，也是本届政府任期的收官之年。当前，淮北正处于转型发展的关键期，既面临许多机遇，又面临不少困难。

    从有利方面看，尽管世界经济总体形势严峻复杂，经济复苏的不稳定性不确定性上升，但全球经济一体化的格局没有改变，我国经济长期向好的趋势不会改变，这为我们转型发展创造了稳定的外部环境。国内经济结构调整步伐加快，经济增长向内需拉动转变，生产要素流动、产业分工转移将加速向中西部地区推进，国家实施积极的财政政策和稳健的货币政策，宏观调控政策的取向是保持经济平稳较快发展，调整经济结构，管理通胀预期，这为我们全面发展开辟了广阔空间。国家《促进中部崛起规划》全面实施，支持资源型城市转型力度加大，省委、省政府更加重视支持皖北发展，三区和濉溪县部分镇被列为省重点开发区域，这为我们科学发展提供了有力支持。经过多年努力，我市工业化、城镇化、城乡一体化加速推进，园区建设初见成效，转型发展的基础逐步夯实。特别是通过一系列的改革创新，立足市情发展的思路更加符合实际，发展的内生动力和提速效应显著增强。

    从不利情况看，欧美实体经济危机开始显露，新兴经济体面临增速放缓和通货膨胀的双重压力，国际市场需求依然疲弱，我国出口增速明显放缓，经济增长下行压力和物价上涨压力并存，部分企业生产经营困难，经济金融等领域也存在一些不容忽视的潜在风险；货币政策虽有微调，但总体偏紧的金融信贷形势仍会持续，特别是土地控制将更严、环保要求将更高。环顾全省，各地竞相发展，我市争先进位的压力越来越大。

    综合分析，做好今年的经济社会发展工作，必须加强战略谋划，增强应对能力，扬长避短，趋利避害；必须树立过“紧日子”的思想，强化打基础、增后劲的意识，深刻认识我市转型发展的阶段性、特殊性，深刻认识转型发展的艰巨性、长期性，坚定信心不动摇，主动作为不折腾，更加积极地抢抓机遇，更加有效地破解难题，努力在新一轮竞争中实现异军突起。

    今年政府工作的总体要求是：坚持以邓小平理论和“三个代表”重要思想为指导，深入贯彻科学发展观，全面落实党的十七届五中、六中全会和省第九次党代会、市第七次党代会精神，牢牢把握转型发展、全面发展、科学发展这一主线，牢牢把握扩大内需这一战略基点，牢牢把握发展实体经济这一坚实基础，牢牢把握改革创新这一强大动力，牢牢把握保障和改善民生这一根本目的，组织开展“转型攻坚年”活动，坚定不移地推进大投入、大招商、大建设、大发展，稳中求进、进中求好、好中求快，保持经济社会平稳较快发展，为争当皖北崛起排头兵、跻身全省第一方阵打下坚实基础。

    综合考虑各方面因素，2012年全市经济社会发展的主要预期目标是：地区生产总值增长12%以上；财政收入增长18%；规模以上工业增加值增长15%；固定资产投资增长25%；社会消费品零售总额增长17.5%；城镇居民人均可支配收入增长14%；农民人均纯收入增长14%；城镇登记失业率控制在4.2%以内；节能减排完成省政府下达的目标任务；人口自然增长率控制在省下达指标以内。

    为实现上述目标，将切实抓好十项重点工作。

    （一）大力实施工业强市战略，推进工业结构优化升级。进一步增强工业的首位意识，扎实开展“五大行动”，着力调优存量、培植增量、壮大总量、提升质量，加快构建煤电和非煤“双千亿”板块。

    实施煤电产业助推转型行动。坚持依托煤、延伸煤、超越煤，支持淮北矿业集团、皖北煤电集团提高在淮投资比例，确保袁店、青东等矿井全面达产，新增原煤产量480万吨。支持淮海实业集团做大做强，进入百亿元大企业行列。推动煤电一体化发展，完成电力投资38亿元以上。打造煤焦化、煤电材、煤电化硅、煤焦电铸产业链，建设煤化工产业基地和国家循环经济发展示范基地。

    实施非煤产业优化升级行动。大力发展方便速食、保健休闲、药食同源等高附加值产品，推进产业集聚，打造中国食品名城。重点发展矿山安全设备制造，提高机械制造集成、配套能力，建设全国重要的煤矿机械制造基地。加快华孚色纺、维科印染退城进区，构建纺纱—织造—印染—服饰配套产业链。积极引进中建材等知名企业，发展新型建材业。力争非煤产业占全市工业总产值的比重提高5个百分点。

    实施新兴产业培育扩张行动。制定实施新兴产业发展规划，用好1亿元专项扶持资金，重点发展新能源、新材料、高端装备制造、节能环保等新兴产业。扩大美信铝基材料、天象石头造纸等新材料产能，发展壮大山河矿装、龙波电气等企业，培育中润生物质能源、太阳能、风能等新能源产业，鼓励安徽天正科技、浙江金龙控股等企业发展电子信息产业，年内开工建设新兴产业项目17个，新兴产业总产值达到160亿元，增长30%。

    实施规模企业提质扩量行动。出台促进企业上规模、上台阶政策措施，开展“中小企业上规模培育年”活动，年内新增规模以上企业100家以上，主营业务收入超亿元企业60家、超10亿元企业8家；力争“十二五”末，全市规模以上企业达到1000家，亿元以上企业500家，10亿元以上企业50家，100亿元以上企业5家，高新技术企业总数超过50家。积极推动企业上市，年内5家企业完成股改，淮矿股份、口子酒业成功上市。推进企业品牌战略和管理创新，鼓励企业更新装备、优化工艺、提高产品能级，新增一批著名商标、名牌产品和省级企业技术中心。

    实施工业运行提速增效行动。深入开展“百名干部驻点帮扶百家企业”活动，落实小微企业减免奖补政策，积极组织企业参加各类产品促销活动，帮助中小微企业解决生产经营中的难题。抓好工业生产运行，做好综合协调保障，建立产业预测预警机制，以月保季、以季保年，确保全年规模工业增加值突破480亿元，力争主营业务收入、利税增幅高于全省平均水平。

    （二）高度重视“三农”工作，统筹推进城乡协调发展。坚持以农民增收为核心，坚持工业化、城镇化和农业现代化同步发展，大力促进农村发展、农业增效、农民增收。

    调整优化农业结构。按照“南粮、北菜、东部经果林”的产业布局，隋堤以南重点发展粮食生产和规模养殖，培育建设30万亩吨粮田；隋堤两侧重点发展瓜菜间作套种、中药材，建立5万亩高效经济作物种植区。北部优先抓好渠沟、朔里等10个重点乡镇的设施精品蔬菜基地建设。东部沿山地带重点发展以石榴、核桃、枣为主的经果林，延伸石榴产业链。相山区重点发展花卉苗木产业，筹建花卉苗木交易市场。继续开展“小麦千斤市”创建活动，实施玉米振兴计划，推动畜禽养殖向边远乡镇布局，培育标准化规模养殖小区50个。加强农业技术推广体系、农产品安全监测体系、动物疫病防控体系建设。按照有偿、自愿原则，通过抓点示范，加快土地流转，力争全年新增土地流转面积10万亩。

    大力促进农民增收。按照“十二五”末农民收入过万元的目标，以村为单位、乡镇为平台，实施农民收入倍增计划。推进农业产业化经营，重点扶持120家农业产业化龙头企业，建设2个农业产业化示范区。规范农村专业合作经济组织，重点培育40家市级农民专业合作社示范社。完善农村集体林权制度改革，积极探索农民住房抵押贷款。落实各项强农惠农富农政策，切实减轻农民负担。继续开展政策性农业保险，分散和化解农业生产风险。

    深入推进城乡一体化。加快临涣、石台等中心镇基础设施建设，建好一批城乡一体化试点镇。大力推进段园、百善等乡镇工业集中区建设，实现产业集聚发展。加快农民集中区建设，新建集中居住区18个以上。加快农村道路等基础设施建设，改造农村公路76公里、危桥35座。加快农村电网升级改造。加强农田水利基本建设，新打机井4015眼，新建涵闸13座、灌溉排涝站27座；新建绿色长廊70公里，完善农田林网10万亩。继续实施农村饮水安全工程，解决10万农村人口安全饮水问题。加快户籍制度、土地管理、社会保障和农村产权制度改革，努力破除城乡一体化发展的体制性障碍。

    （三）力促服务业优化升级，快速扩大产业规模总量。落实全省服务业发展会议精神，坚持市场化、产业化、社会化导向，加快培育具有核心竞争力的支柱产业，打造皖北能源、煤化工及农产品配套服务基地。

    改造提升传统服务业。抓好淮海建材大市场、大润发二期等续建项目建设, 开工建设温哥华城五星级酒店和乐天玛特大卖场，建设时尚购物、高端餐饮、美食小吃等特色商业街。加快红星美凯龙、中泰广场二期、兆基商业广场、大淮海工程机械城等专业市场建设。继续推进“新网工程”、“万村千乡工程”，完善城乡现代流通服务体系。推进房地产业稳健发展，促进房地产业与经济、民生协调发展。

    大力发展文化旅游业。修编《淮北市“十二五”旅游业发展规划》，依托山水资源和人文特色，大力发展休闲旅游、农家乐旅游和文化旅游，努力把淮北建成区域性旅游休闲城市。加快国购汽车主题综合体、南湖光影文化传播基地、口子文化产业园和酒文化博物馆、儿童梦幻王国、洪庄文化产业园、舒伦仕颐养生态社区等文化旅游项目建设。建成龙脊山、塔山农家乐旅游项目一期工程。强化旅游目的地功能构建和形象提升，推出特色旅游线路，加大旅游宣传推介力度，力争旅游业发展实现新突破。

    积极发展现代服务业。围绕新型工业化和现代化进程，加快发展物流服务业，提升蓝宇、百路物流基地运营能力，开工建设淮矿现代物流产业园，筹划食品物流园区和段园物流园区。突出产业引领，培育发展服务外包、电子商务、法律服务、文化创意、节能环保、健康养老等新兴服务业。整合会展资源，办好2012年中国（淮北）煤矿机械博览会和首届中国（淮北）食品工业博览会。

    （四）提高城市综合承载能力，奋力打造山水生态新城。继续扎实开展文明城市创建活动，不断提升城市功能和品质，打造“皖北江南、文明淮北”。

    继续完善提升城市功能。实施《淮北市城市近期建设规划（2011-2015年）》，改造提升老城区功能，推动东部新城建设，加快相濉、相烈、相杜一体化进程。建设淮海东路、东山北路、梧桐南路，改造濉溪路、淮海路，加快推进境内萧淮路南北段改造。开工建设市党校新校区，加快新火车站、市灭火救援指挥中心、市公安局监管中心建设。完成幸福巷、康复巷、建安路等老城区街巷和鹰山中路、长山中路、相山中路、孟山中路、南黎路等城区主干道路综合改造提升工程。推动城市均衡发展，引导老城区优质公共资源向新城区转移。

    扎实推进生态淮北建设。积极探索新型工业化、城镇化与生态化融合发展，突出“山、水、湖、绿”，显山露水，逐步形成“两山环绕、六湖珠连、城在山中、水在城中”的山水生态城市框架。加快相阳沟、跃进河综合整治和东湖治理。计划投资2500万元，绿化新建道路20公里。投资3000万元，实施城市主要出入口和城区绿化提升改造。投资2400万元，完成东部荒山1万亩绿化任务，栽植各类苗木100多万株。扩大农村清洁工程试点范围，新增4-8个试点乡镇。大力推进节能减排，严格执行重点行业环评、能评准入和污染排放标准，加快一批节能技改工程、污水处理设施建设，培育一批循环型企业和生态园区，淘汰一批落后产能，全面完成省下达的节能减排年度控制目标。

    继续推进文明城市创建。坚持“创建为民、为民创建”，把文明城市创建作为永不竣工的民生工程，持之以恒地加以推进。围绕城市公共文明指数测评，深入开展公共环境、公共秩序、公共设施治理活动。围绕社会主义核心价值体系建设，深化公民思想道德建设，持续开展道德模范、“身边好人”评选及各类志愿服务，加强社会诚信体系建设，不断提升市民文明素质。创新城市建设管理，探索包容性管理新模式、新方法、新领域，启动新一轮社区改造工程，推进小区物业属地化管理，进一步完善社区服务功能，不断夯实创建工作基础，让广大市民更多地享受到文明创建成果。

    （五）强力推进重点项目建设，着力增强经济发展后劲。抓项目就是抓发展、抓大项目就是抓大发展、抓好项目就是抓科学发展。全面实施“项目建设推进年”活动，以项目推动产业升级，以项目促进跨越发展。

    千方百计谋划争取项目。完善项目谋划生成机制，调整充实重大项目谋划争取领导小组和办公室，重点在传统产业、现代服务业、战略性新兴产业和重大基础设施等领域，谋划、包装、储备一批投资规模大、产业链条长、事关长远发展的重大项目。全年谋划重大项目100个，总投资超600亿元。加大项目争取力度，做好项目申报前期工作。继续争取国家对资源枯竭型城市的政策扶持，力争更多项目列入国家和省投资计划。

    集中精力加快项目建设。年内新建、续建重点项目232个，完成年度投资330.6亿元。积极推进淮水北调、浍河治理、符夹线改造等项目前期工作，开工建设平山电厂、口子工业园等重点项目，扩大宝迪集团、神华肉食、安徽机电、富士特铝业等企业产能，加快临涣煤焦化二期、鸿源煤化工、天悦工贸、华孚等项目建设进度，力争大唐虎山电厂、220千伏铚城输变电工程等项目主体工程完工。

    全力以赴抓好项目调度。进一步完善项目月调度、季观摩制度，实行市级领导联系项目、督查督办项目制度，建立协调调度事项跟踪落实和激励机制，将时间节点、目标任务分解落实到责任单位，定人定岗定时完成。加强项目知识培训，提高谋划、建设、管理的能力。扎实做好项目开工前的立项、规划、环评、用地等前期工作，建立重点项目审批绿色通道，强化项目动态管理，确保项目早开工、早建成、早投产。

    （六）坚持招商引资第一要事，全面激发经济发展活力。立足内联外引、招大引强，不遗余力推进招商引资工作。

    创新招商方式方法。坚持领导带头招商、招商分局驻点招商和小分队专业招商相结合，充分发挥市、县区开发区招商引资的主体作用，进一步加强与央企、民企对接，开展专题招商、产业链招商和大项目定点招商，着力引进投资额度大、科技含量高、产业关联度强的基地型、龙头型项目，宽领域、多层次、全方位招商引资。力争全年引进超亿元项目50个以上，引进资金突破500亿元。

    健全招商工作机制。加强招商引资工作市级统筹，健全招商信息筛选、项目评估、政策把关、签约落地机制，专人负责、跟踪服务，力争瑞典欧特利、天津康师傅、开诚机器人、三一重工等在谈项目尽快签约立项、开工建设。继续推行“六个一”工作机制，建立签约项目建设限时制、跟踪服务问责制和项目开竣工保证金制，完善招商绩效评估和考核机制，以硬约束倒逼硬指标，完成硬任务。

    加强招商载体建设。抓住省推动开发区转型升级的重大机遇，促进园区整合，争取4个省级开发区筹建区成为省级开发区。强力推进市开发区新区建设，加快实施二期14.74平方公里“九通一平”，确保梧桐大道、吉山路、滨河路等主次干道全面建成通车，一期31万平方米安置房交付使用，二期59万平方米完成主体工程。全面推进龙湖工业项目区北部3.2平方公里的道路和管网配套，力争年内市经济开发区实现产值160亿元、财政收入7亿元。加快临涣工业园基地北路等基础设施建设，抓好煤焦化综合利用、延伸加工，积极争创国家循环经济示范园。同步推进各县区园区基础设施建设，完善金融网点、担保公司、公租住房等服务配套，年内各扩展3平方公里以上。

    （七）持续加大科技创新力度，不断提升产业竞争优势。坚持科技兴市不动摇，扎实推进国家技术创新工程试点工作，着力构建以企业为主体、市场为导向、产学研紧密结合的技术创新体系，助推产业升级、城市转型。

    积极搭建“三个平台”。搭建企业科技孵化平台，编制实施科技园区创建3年行动方案，依托市经济开发区、濉溪经济开发区，建设科技孵化器，争创国家级高新技术开发区；依托凤凰山开发区，建设国家级食品科技园区；依托杜集开发区，争创国家级矿机装备高新技术产业基地；依托烈山区，争取立项建设国家可持续发展试验区。搭建产学研合作平台，加强与高校、央企、科研院所合作，开展“百名教授百企行、百家企业联院校”活动，在矿山机械装备、铝基材料等领域，组建1-2个产业技术创新战略联盟。搭建企业技术研发平台，加快企业研发机构建设，新增省级企业技术中心4家、省级工程技术研究中心3家；推进院士和博士后科研工作站建设，筹建粉煤灰、煤矸石及秸秆综合利用、现代食品加工技术研究院。

    着力实施“三大工程”。实施新兴产业科技引领工程，在新能源、新材料等领域引进实施一批科技项目，开发一批具有自主知识产权产品，力争新增市级以上创新型试点企业10家，省级以上重点新产品5-7个，专利申请量翻一番，达到1600件以上。实施高新技术企业培育工程，开展“高新技术企业培育年”活动，实现高新技术企业和高新技术产品双倍增目标，高新技术产业总产值达到130亿元。实施“小巨人”培养工程，培育一批技术含量高、市场前景好的科技型企业，力争认定市级科技“小巨人”企业50家。

    （八）努力破解发展要素制约，提升可持续发展能力。金融是现代经济的命脉，土地是财富之母，人才是第一资源。坚持以改革的思路、创新的思维、开放的手段，重点破解资金、土地、人才三大瓶颈制约，保障经济社会平稳较快发展。

    继续深化金融创新。支持企业发行债券和中期票据，不断拓宽融资渠道。争取国家农发行、徽商银行、浙江泰隆商业银行在淮设立村镇银行，完成濉溪县农村信用联社改制。支持各银行业金融机构向上争取规模，加强银企对接，确保新增贷款增速不低于全省平均水平。积极推进金融产品创新，推行农户信用联保、金融仓储等信贷新业务。筹建担保行业协会，做大做强小额贷款公司和融资性担保公司。创新保险资金使用方式，争取保险资金以股权、债权等方式投资我市重大工程项目。加强政府投融资平台建设，整合存量资产，扩大资本规模，提高信用等级，规范资金运营管理，增强投融资能力。

    依法依规集约节约用地。积极通过单独选址、点供、增减挂钩等方式争取用地指标。抓住我市作为全省唯一工矿废弃地利用试点市的机遇，用好盘活包括塌陷地在内的工矿废弃地。坚持有保有压，优先保障市开发区新区、民生工程等建设用地。实行土地预审联审制度，重点加强对各县区、各类园区工业用地的监管，严把入园项目产业政策、投资强度、亩均税收三道关，鼓励企业多建多层标准厂房。进一步加大土地清理处置力度，坚决遏制违法违规用地。

    强化人才支撑作用。实施相城人才引进和产业创新团队计划，围绕我市主导产业，加快培育一批产业创新团队和创新团队带头人，实现产业优势和高层次人才的良性互动。实施企业家素质提升工程，加强企业家战略思维、决策能力和领导能力培训。主动适应转变经济发展方式的新要求，及早谋划调整人才专业素质结构、层级结构和分布结构，打破阻碍人才流动的体制性障碍。开展“淮北市优秀人才”、“优秀企业家”、“创业之星”等评选活动，努力营造鼓励脚踏实地、勤劳创业、实业致富的社会氛围。

    （九）持续加强民生工程建设，促进社会事业协调发展。坚持把惠民生、促和谐放在政府工作的首位，扩大和改善公共服务，加快发展社会事业，让广大人民群众共享发展成果。

    全面实施民生工程。进一步优化财政支出结构，把财力更多地投向民生工程。积极推进城镇居民医疗保险市级统筹和门诊费用统筹，提高医疗保险待遇水平。提高农村五保集中供养率，力争集中供养率达到50%。继续实施农村敬老院改扩建工程。建成市社会福利中心和救助站。建成安置房152万平方米，开工建设6000套公共租赁住房、8000套棚户区改造住房。实施好法律援助工程。加强民生项目管理和考核奖惩，不断提高民生工程的质量和效益。

    加强就业和社保工作。重点抓好高校毕业生、城镇就业困难人员和农民工就业，全年新增就业3.5万人。扎实开展创业型城市创建活动，实施“小老板”工程，加快建设刘桥、临涣等11个省级农民工创业园，以创业带动就业。切实维护劳动者合法权益，构建和谐劳动关系。完善社会保障体系，扩大社会保险覆盖面，落实被征地农民养老保险政策，开展城镇居民社会养老保险试点。健全城乡居民最低生活保障制度，完善社会救助体系。

    繁荣发展文化事业产业。全面贯彻党的十七届六中全会精神，推进文化强市建设。加大文化基础设施建设力度，谋划建设淮北大戏院、汉画像石馆和美术馆。完成市豫剧团、歌舞团改制工作。组织实施“文化精品工程”，推出更多群众喜闻乐见的文艺作品。加快一批文化产业项目建设，构建休闲、创意、旅游、科技、影视五位一体的文化产业发展新格局。推动大运河保护和申遗，启动柳孜码头遗址二期发掘工作。深入开展“扫黄打非”活动，加大文化市场整治力度，坚决打击各种侵权盗版行为。

    大力发展教育卫生事业。积极发展学前教育，新建幼儿园13所，改建、扩建公办幼儿园36所。均衡发展义务教育，推进义务教育学校标准化和农村薄弱学校建设；完成市一小南校区建设，做好市一中东校区建设前期工作，加快市二中、梅苑学校集团化办学进程。创新发展职业教育，落实职业教育扶持政策，开工建设总投资15亿元的市职业教育园区。推动东部大学城加快建设。巩固基层医疗卫生体制改革成果，实现新型农村合作医疗制度全覆盖，适时推进公立医院综合改革。做好卫生应急和重大传染病防治工作。建立市级医疗紧急救援指挥中心，开工建设市妇幼保健院二期工程。

    统筹发展其他各项社会事业。深入开展全民健身运动，筹办好市第九届运动会。加强人口和计生工作，综合治理出生人口性别比，做好流动人口计生管理与服务。启动老干部服务中心建设。稳妥推进事业单位分类改革。关注残疾人事业发展。加强信息化条件下的保密工作。加强国防教育和国防后备力量建设，大力支持驻淮部队建设，进一步加强国防动员、民兵预备役和人民防空工作，扎实做好双拥安置优抚工作，营造军政军民团结的良好氛围。加强统计力量，推行“在地统计”和“企业一套表”制度改革，完善服务业统计。抓好机关事务、住房公积金、档案、地方志工作，推进外事侨务、对台、民族宗教、贸易促进、地震、气象、红十字会工作。重视和推动妇女儿童、老龄、社会福利、慈善等事业发展，支持工会、共青团、妇联等人民团体参与社会管理和公共服务。

    （十）深入推进社会管理创新，全力维护社会和谐稳定。完善应急管理体系，提高应对处置各种突发事件能力。加强物价监测预警，保持市场价格总水平基本稳定。抓好以群众工作统揽信访工作试点，深化大走访活动成果，落实各级干部接访、下访、回访和联系群众制度，着力化解一批信访积案。加强社会矛盾纠纷排查调处和预警体系建设，努力把矛盾和问题消除在萌芽状态。全面完成开放式村级组织服务用房建设。深入开展“六五”普法。扎实推进“平安淮北”建设，深入开展“打四黑除四害”专项行动，严密防范、依法打击各种违法犯罪活动，为人民群众创造安全、稳定、有序的工作生活环境。大力开展交通、消防特别是建筑领域的专项整治，抓好煤矿、非煤矿山、危险化学品等重点领域的安全监管，严防各类安全生产事故发生。强化产品质量安全、食品安全监管和药械市场监督检查，严肃查处产品质量安全违法违规案件。

****三、政府建设：着力提升服务发展能力****

    目前，淮北正处于转型发展的关键时期，改革、发展、稳定面临新的形势，必须进一步加强政府自身建设，弘扬职业、专业、敬业精神，不辜负人民的新期待。

    （一）进一步强化责任意识，开拓创新、奋发有为。担当的责任意识应该体现在锐意改革、积极创新、敢作敢为的工作干劲上，应该体现在对工作负责、对人民诚信的积极态度上。对正确的决策部署，要敢于坚持，一抓到底；在关键时刻、紧要关头，要敢于负责、敢于决断、敢于碰硬；在问题和困难面前，要敢于正视，不回避、不退缩。坚持以业绩论英雄、无功便是过，进一步激发和调动方方面面的创造力、凝聚力和执行力。凡是向群众承诺的事情，要创造一切条件，克服一切困难兑现，做到承诺一件，兑现一件，确保干就干成，干就干好。

    （二）进一步强化服务意识，增强本领、提升能力。“权力就是责任，工作就是服务，公务员就是服务员”。坚持把学习作为一种责任来履行，把服务作为一种准则来遵循，大力推动学习型、服务型政府建设。切实服务好群众，牢固树立群众利益无小事的思想，深入基层，深入群众，把更多的时间和精力用在帮助群众解决实际困难和问题上；珍惜民力，尊重民意，把有限的财力用到经济社会协调发展和提高人民生活水平上。切实服务好企业，全面推行为企服务全程代理制，以真策实招帮助企业解决生产经营中的难题，坚决杜绝“吃、拿、卡、要”行为，为企业发展营造良好的环境。切实服务好基层，继续简政放权，在市级权限范围内，扩大县区经济社会管理权限。

    （三）进一步强化效率意识，敢干会干、实干快干。着力优化政府工作流程，深化行政审批制度改革，全面推行并联审批、联合办公、网上办公与现场解决问题等工作制度。雷厉风行抓落实，做到件件有着落、事事有回音。牢固树立全市工作“一盘棋”思想，自觉加强工作协同，对工作拖拉、推诿扯皮、影响大局的，予以严肃查处，公开曝光。全面开展政府绩效管理工作，建立健全绩效管理评估办法，强化绩效考评结果运用，不断提升执行力和公信力。

    （四）进一步强化法治意识，依法行政、公开透明。着力规范行政执法行为，强化执法监督，推进公正执法、文明执法。规范行政处罚自由裁量权，强化行政复议工作。建立健全行政审批责任制度，强化行政审批的全过程监控。加强政务公开，健全科学民主决策机制，重大政策、重大项目和民生事项坚持专家论证、社会公示或公开听证制度，凝聚各方智慧，提高政府决策科学化、民主化水平。各级政府及其工作人员都要自觉接受人大及其常委会的法律监督、工作监督和政协的民主监督，认真办理人大代表建议议案和政协委员提案，认真听取民主党派、工商联、无党派人士和人民团体的意见建议，主动接受社会公众和新闻舆论监督。

    （五）进一步强化廉政意识，自律清廉、争做表率。加强反腐倡廉制度建设，建立健全对领导干部、人财物管理使用、关键岗位的监督和制约机制，全面推行廉政风险防控管理工作，努力从源头上预防和治理腐败。完善全市集中统一的招标采购监管体制，规范招标采购行为，加强标后合同管理。加强对重大投资项目实施的监督检查，严格执行经济责任审计、重大事项报告等制度。加强警示教育，促进政府全体工作人员自警自励、勤政廉政。严肃查处损害群众利益的案件，以反腐倡廉的实际成效凝聚民心、推动发展。

    各位代表，艰辛成就伟业，奋斗创造辉煌。让我们在中共淮北市委的坚强领导下，团结带领全市人民，抢抓机遇、奋力赶超，全面完成今年的各项目标任务，努力在转型发展中快速崛起，以优异成绩迎接党的十八大胜利召开！
